# Receptive Vocabulary, Phonological Short-Term Memory, Theory of Mind and Oral Inferential Comprehension in French-Speaking Preschoolers With and Without Developmental Language Disorder

**DOI:** 10.1177/23969415251353154

**Published:** 2025-06-20

**Authors:** Pamela Filiatrault-Veilleux, Julia Pichonsky, Chantal Desmarais

**Affiliations:** 1 Department of Communication Sciences and Disorders, Faculty of Rehabilitation Medicine, 3158 University of Alberta, Edmonton, Alberta, Canada; 2 School of Rehabilitation Sciences, Faculty of Medicine, 4440 Université Laval, Quebec City, Quebec, Canada; 3 Center for Interdisciplinary Research in Rehabilitation and Social Integration (CIRRIS), Quebec City, Quebec, Canada

**Keywords:** Developmental Language Disorder, inferential comprehension, listening comprehension, receptive language, theory of mind

## Abstract

**Background and aims:**

Inferential comprehension difficulties and their impacts on reading comprehension are well documented in school-aged children with Developmental Language Disorder (DLD). In comparison, fewer studies have been conducted in young children with DLD prior to their formal schooling journey. In addition, the contribution of linguistic and cognitive skills to oral inferential comprehension abilities in preschoolers, notably receptive vocabulary, phonological short-term memory, and theory of mind (ToM), requires further investigation. The first aim of this study is to explore how young children presenting with DLD aged 5 to 6 years perform when compared to same-age and younger children presenting with typical language development (TLD) on measures of oral inferential comprehension, receptive vocabulary, ToM, and phonological short-term memory. The second aim is to analyze how these linguistic and cognitive skills contribute to oral inferential comprehension in both DLD and TLD preschool children.

**Methods:**

A total of 112 preschool children participated in this study, including 21 (*n* = 21) children with DLD aged 5 to 6 years and two TLD groups, 37 (*n* = 37) younger children aged 4 to 5 years and 54 (*n* = 54) same-age children. A narrative-based oral inferential comprehension task was administered to all children, in addition to measures of receptive vocabulary, phonological short-term memory, and ToM. Analysis of covariance (ANCOVAs) were used to compare performances between the three groups, followed by Pearson correlations and hierarchical regression analyses to examine the contribution of these variables to oral inferential comprehension abilities across the sample.

**Results:**

After controlling for level of parental education (LPE) and biological sex, children with DLD performed significantly below the same-age TLD group on all four measures with large effect sizes (*p* < .001; *η*^2^ = .17–.44). Their performance was comparable to the younger TLD group on measure of oral inferential comprehension (*p* = .234), and significantly below on measures of receptive vocabulary (*p* = .008), phonological short-term memory (*p* < .001), and ToM (*p* = .028). Results from the regression analysis indicated that age, LPE, diagnosis condition, receptive vocabulary and ToM accounted for 53% of the total variance in oral inferential comprehension.

**Conclusions and implications:**

This study reiterates the early listening comprehension difficulties experienced by preschool children with DLD when compared to children presenting with typical language development. The results also indicate that when controlling for age, LPE and diagnosis condition, children are likely to have better inferential comprehension abilities if they perform well on a measure of ToM. Considering that challenges related to language comprehension are acknowledged to be persistent and less responsive to intervention, these findings can help inform the development of evidence-based interventions aiming at supporting language comprehension of young children with DLD.

## Introduction

Developmental Language Disorder (DLD) is a lifelong neurodevelopmental condition that is characterized by significant deficits in language learning, comprehension, and expression with functional impacts on everyday interactions and/or educational outcomes (Bishop et al., 2017). Prevalence studies estimate that DLD affects about 7% of children (Tomblin et al., 1997; Norbury et al., 2016), making it one of the most prevalent neurodevelopmental disorders. Yet, DLD remains under-recognized with limited access to research funding and clinical services (Kulkarni et al., 2022; McGregor, 2020). Importantly, when compared to peers with typical language development (TLD), it has been shown that DLD is associated with persistent depressed academic achievement (Tomblin, 2008), lower vocational qualifications (Conti-Ramsden & Durkin, 2012) and increased social, emotional, and behavior challenges (Dubois et al., 2020; Yew & O'Kearney, 2013). Of note, Law et al. (2009) carried out a longitudinal survey with a DLD cohort from age 5 to 34, finding that poor reading, mental health challenges, and higher risk for unemployment are associated with long-term outcomes for these individuals. As a result of all these associated risks, DLD is considered a public health concern (Law et al., 2013; Reilly & Mckean, 2023). Therefore, assessing and screening for language difficulties early on is critical, with the added imperative of providing sustained, effective early support for young preschool-aged children to decrease risk for these aforementioned associated outcomes in adolescence and early adulthood (Reilly & Mckean, 2023).

In particular, research indicates that difficulties with listening comprehension in children with DLD are persistent and often remain into adulthood (Hogan et al., 2014). Challenges with listening comprehension have been shown to be less responsive to intervention compared to expressive language, in addition to current limited evidence supporting the efficacy of oral language comprehension interventions (Acosta-Rodríguez et al., 2022; Clark et al., 2007; Donolato et al., 2023; Roberts & Kaiser, 2012; Tarvainen et al., 2020; Law et al., 2003). Moreover, identifying listening comprehension difficulties in this population can be difficult, particularly if they occur in isolation, and there are still limited effective assessment tools to screen for these challenges (Alonzo et al., 2016; Nation et al., 2004; Wittke & Spaulding, 2018). Hence, the development of evidence-based practices supporting listening comprehension abilities in individuals with DLD have been highlighted as key priorities for future research (Kulkarni et al., 2022). In light of these challenges, there is a need for research that focuses on the development of sensitive tools and effective intervention programs rooted in scientific research that provides a better understanding of early listening comprehension profiles of young children with DLD from a young age.

### Inferential Comprehension in Preschool Children With and Without DLD

Listening comprehension, defined as the ability to understand spoken words, sentences, and discourse, can be divided into two levels: literal and inferential comprehension (Bishop, 2014; Paul, 2000). Literal comprehension refers to the understanding of explicit information, such as individual words and sentences (Kendeou et al., 2020; van Kleeck et al., 2006). In contrast, inferential comprehension requires the ability to interpret a message in a context where some information is not directly stated (Dawes et al., 2018, 2019; Filiatrault-Veilleux et al., 2015a, 2016b, 2024; Gineste & Le Ny, 2002; van Kleeck, 2008). For example, a child who hears their mom saying “Jasmine, please bring your umbrella to school today!”, will understand that they need to bring their umbrella to school (literal comprehension), because it is raining outside (inferential comprehension). Therefore, while interpreting words and grammar, a child must simultaneously construct a mental representation of the other person's message, using, among other things, theory of mind (ToM) skills, defined as the ability to infer others’ mental states, such as beliefs, desires, or intentions (Baron-Cohen et al., 2000; de Villiers & de Villiers, 2014). To achieve this, the child must fill in the nonexplicit gaps by establishing links between what is being said and the context, while referring to their own world knowledge. Inferential comprehension is therefore defined as the ability to integrate linguistic knowledge (words and grammatical rules) with social and contextual knowledge, in order to create a complete and coherent mental representation of a message (Kendeou et al., 2020; van Kleeck, 2008).

Inferential comprehension has been shown to be essential to the understanding of many messages during early childhood (starting at 2–3 years of age), particularly in the context of aurally presented narratives and social interactions (e.g., Filiatrault-Veilleux et al., 2015a, 2016b; Kendeou et al., 2020; van Kleeck, 2008; Makdissi & Boisclair, 2006; Ford & Milosky, 2003), and continues to be essential for reading comprehension during the school years (Botting & Adams, 2005; Ford & Milosky, 2003; Spackman et al., 2006; Silva & Cain, 2015; Cain & Oakhill, 2007; Spencer et al., 2014; Botting & Adams, 2005; Kendeou et al., 2008; van Kleeck, 2008). Research into the inferential comprehension abilities of children with DLD has consistently revealed significant challenges when compared to their typically developing peers (Dawes et al., 2018, 2019; Filiatrault-Veilleux et al., 2015b; Adams et al., 2009; Ford & Milosky, 2008; Bishop, 2014). This is important because difficulties in inferential comprehension can impact academic performance in activities such as following instructions, engaging in classroom discussions, and reading comprehension that rely heavily on inference making (van Kleeck, 2008; Cain & Oakhill, 2007). Furthermore, these challenges can extend to social interactions and emotional development (Conti-Ramsden & Durkin, 2012; Ford & Milosky, 2003) given that understanding social cues and participating in peer conversations also depend on inferential comprehension abilities. Consequently, young children with DLD may face cascading effects of inferential comprehension difficulties on both their academic success and social-emotional wellbeing.

#### Linguistic and Cognitive Skills Contributing to Inferential Comprehension Abilities in Preschool Children With and Without DLD

Inferential comprehension is a complex, higher-order skill that relies on a range of language and cognitive abilities (Cain & Oakhill, 2007; Currie & Muijselaar, 2019; Kim, 2016, 2020). Previous research has highlighted receptive vocabulary as a key predictor of inferential comprehension (Sterpin et al., 2021). Silva and Cain (2015) found that, among 82 TLD English-speaking children aged 4 to 6, receptive vocabulary was the only significant predictor of oral inferential comprehension after controlling for age and nonverbal ability. Similarly, Florit et al. (2011) reported that receptive vocabulary and semantic knowledge accounted for a significant amount of variance in both literal and inferential comprehension of aurally presented stories in a study of 221 Italian-speaking children of the same age range. Expanding on these findings, Potocki et al. (2012) examined 131 TLD French-speaking children and found that verbal short-term memory, vocabulary, sentence comprehension, grammatical judgment, and morphological knowledge explained 44% of the variance in literal and inferential narrative comprehension. Additionally, ToM has been identified as an important contributor to language comprehension (Dawes et al., 2018; Jackson et al., 2022; Guajardo & Cartwright, 2016). For instance, when investigating the longitudinal effects of ToM on listening comprehension in TLD children aged 3 to 6, Jackson et al. (2022) found an indirect concurrent effect of ToM on later listening comprehension. Altogether, these findings suggest that verbal short-term memory, vocabulary, semantic knowledge, grammar, and ToM may be all important predictors of inferential and literal narrative comprehension in TLD children aged 4 to 6 years (Silva & Cain, 2015; Florit et al., 2011; Potocki et al., 2012).

While inferential comprehension difficulties and their impact on reading comprehension are well-documented in school-aged children with DLD (Adams et al., 2009; Bishop, 2014), research on younger children with DLD, prior to formal schooling, remains limited (Dawes et al., 2018; Ford & Milosky, 2008; Filiatrault-Veilleux et al., 2015b). Investigating linguistic and cognitive predictors of inferential comprehension in preschool-aged children with DLD, Dawes et al. (2018) found that both narrative macrostructure and ToM were positively associated with inferential comprehension in 5- to 6-year-olds. While vocabulary was a significant factor in a bivariate regression analysis, it was not in a multiple regression analysis, suggesting that children with DLD may rely less on vocabulary for inference-making compared to their TLD peers. In contrast, studies on school-aged children with DLD have identified vocabulary knowledge (Botting & Adams, 2005), grammatical knowledge (Botting & Adams, 2005), sentence comprehension, verbal short-term memory (Karasinski & Ellis Weismer, 2010; Dodwell & Bavin, 2008), and nonverbal IQ (Botting & Adams, 2005) as key predictors of inferential comprehension.

Preschool children with DLD often face challenges with respect to receptive vocabulary (Gray, 2006; Sansavini et al., 2021), ToM (Andrés-Roqueta et al., 2013; Nilsson & Lopez, 2015; Vissers & Koolen, 2016), and phonological short-term memory (e.g., Alt, 2011), which may all contribute to inferential comprehension difficulties both inside and outside the classroom (Dawes et al., 2018; Karasinski & Ellis Weismer, 2010; Dodwell & Bavin, 2008; Potocki et al., 2012; Florit et al., 2011; Silva & Cain, 2015). Despite the well-documented impact of these cognitive and linguistic factors, further research is needed to more clearly understand their interplay in inferential comprehension difficulties among children with DLD. Improved understanding of these linguistic and cognitive skills can inform early intervention approaches that target receptive language and overall supplement the growing body of literature on oral inferential comprehension development in preschoolers with DLD.

Building on the work of Dawes et al. (2018), this study aims to investigate the contribution of ToM, receptive vocabulary and phonological short-term memory on oral inferential comprehension using a large sample size of children with and without DLD. Because differences between children with DLD and same-age TLD peers are expected, the inclusion of a younger TLD group will serve as an important control in the examination of these variables (Leonard & Schroeder, 2023). By incorporating group comparisons (DLD vs. same-age TLD vs. younger TLD) and whole-sample analysis (DLD and TLD), in addition to accounting for confounding variables such as LPE, age, and biological sex, this study will provide a more comprehensive understanding of the early listening comprehension profiles of preschoolers with and without DLD. Identifying these early associations is essential for developing targeted interventions that can support young children with language difficulties before they enter formal schooling.

## The Present Study

The present study aims to describe oral inferential comprehension abilities, receptive vocabulary, ToM, and phonological short-term memory in young French-speaking preschoolers with and without DLD. Our two research questions are:

1. How do young children presenting with DLD aged 5 to 6 years perform when compared to (a) same-age (5–6 years) and (b) younger TLD peers (4–5 years) on measures of oral inferential comprehension, receptive vocabulary, ToM, and phonological short-term memory after controlling for LPE and biological sex?
2. What is the contribution of receptive vocabulary, ToM, and phonological short-term memory to oral inferential comprehension abilities of young preschool children (DLD and TLD) aged 4 to 6 years of age, after controlling for confounding variables such as age and LPE?

## Method

This study received ethics approvals from the Université Laval (#2013-700, #RIS_2013-309) and the University of Alberta ethic boards (#Pro00137609). Caregivers of all participants completed and signed an informed written consent form prior to data collection.

### Participants

A total of 112 French-speaking preschool children aged 4 to 6 years of age were included in this study. Twenty-one (*n* = 21) children aged 5 to 6 years presenting with DLD (mean age = 73.7 months; *SD* = 8.2 months; 14 boys; 4 girls) were recruited in collaboration with francophone Speech-language pathologists from rehabilitation centers and school boards across three provinces in Canada (Quebec, Alberta, and British Columbia). In order to be included in the study, the participants had to meet the following criteria: (1) speak French as their first language; (2) attend a preschool or school where the language of instruction was French; and (3) have received a formal diagnosis of DLD by a registered Speech-Language Pathologist consistent with Bishop et al. (2017) criteria. Children were excluded if presenting with a diagnosis of global developmental delay or diagnosed with a vision or hearing impairment.

The two comparison groups of TLD children consisted of previously collected anonymized data drawn from a larger study aimed at describing the development of inferential comprehension in 3 to 6 years old children (Filiatrault-Veilleux et al., 2016a). The two groups comprised 37 (*n* = 37) younger children aged 4 to 5 years recruited from mainstream daycares and preschool centers (mean age = 56.2 months; *SD* = 6.2 months; 19 boys, 18 girls) and 54 (*n* = 54) same-age children aged 5 to 6 years recruited from mainstream kindergartens (mean age = 73.7 months; *SD* = 3.4 months; 27 boys, 27 girls).

Participants characteristics are presented in Table 1. Of note, the participants significantly differed in terms of biological sex and parental level of education, with more boys in the group of children with DLD (*X*^2^ (2, *N* = 112) = 6.425, *p* = .040) and showing lower parental education levels (*X*^2^ (4, *N* = 110) = 14.827, *p* = .005) when compared to the two typical language groups. These results are in line with research relating lower maternal education levels and biological sex as case history risk factors for DLD through a systematic review and meta-analysis (Rudolph, 2017).

**Table 1.** Participant Characteristics by Groups (DLD vs TLD).

Variable	5-6 y.o. DLD *(n* = 21)	4-5 y.o. TLD (*n* = 37)	5-6 y.o. TLD (*n* = 54)	Group comparisons		
				Test statistic	*p* Value	Post hoc comparisons
Age in months *(M (SD))*	73.7 (8.2)	56.2 (6.2)	73.7 (3.4)	*F*(2,109) = 116.8	<.001	4–5 y.o.TLD < 5–6 y.o. TLD = 5–6 y.o. DLD
Biological sex (% boys)	81.0	51.4	50.0	*Χ*^2^ (2) *=* *6.4*	.040	5–6 y.o. DLD > 4–5 y.o.TLD = 5-6 y.o.TLD
Parental age of responding parent in years (*M (SD))*	35.9 (6.4)	33.6 (4.1)	36.6 (4.8)	*F*(2,108) = 4.1	.019	4–5 y.o.TLD < 5–6 y.o. TLD
LPE (%)						
≤High school	15.8	0	0	*Χ*^2^ (4) *=* 14.8	.005	5–6 y.o. DLD < 4–5 y.o.TLD = 5–6 y.o. TLD
Diploma/certificate	14.3	21.6	20.4			
University	68.4	78.3	75.9			
Income (%)						
≤39 999	23.8	8.1	5.6	*Χ*^2^ (4) *=* 7.4	.116	N/A
40 000–79 999	19.0	18.9	18.5			
>80 000	47.6	74.0	75.9			

*Notes*. DLD = Developmental Language Disorder; LPE = level of parental education; TLD = Typical Language Development; y.o.=year-old.

#### Procedures

All measures were administered by registered practicing speech-language pathologists or supervised graduate students in speech-language pathology as part of a regular 45-minute individualized session at the rehabilitation center or at school. Scoring of tests was completed by two trained research assistants who received training by the research team.

### Measures

#### Inferential Comprehension

An assessment tool entitled *Évaluation de la Compréhension Inférentielle en Récit (É.C.I.R.)* (Filiatrault-Veilleux & Desmarais, 2020) consisting of a dialogic reading task with scripted causal inferential questions was used to assess inferential comprehension (Filiatrault-Veilleux et al., 2016a, 2024). The *É.C.I.R.* is a 20-page long story that follows a predictable narrative structure (i.e., initiative event/problem, internal response, goal, prediction, attempt to solve the problem, and resolution). Using a narrative-based approach, 19 online pre-recorded inferential questions are asked to children in order to assess six causal inference types related to story grammar elements (Filiatrault-Veilleux et al., 2016b). The child's responses obtained to each question are transcribed, then scored using a fine-grained approach according to four categories following a quality continuum ranging from expected to inadequate (3 points = expected, 2 points = incomplete, 1 point = low contingency, 0 point = inadequate or off topic), as described in previous studies (Desmarais et al., 2013; Filiatrault-Veilleux et al., 2016a, 2024). The total raw score is calculated out of 78 points. Psychometrics of the ECIR are reported in a previous study (Filiatrault-Veilleux et al., 2016b). Of note, the concurrent validity coefficient with a previous experimental task of inferential comprehension (Desmarais et al., 2013; Filiatrault-Veilleux et al., 2015b) is of .77 and the convergent validity with receptive vocabulary is .43 (as assessed with the French version of the PPVT-R; Dunn et al., 1993). The fine-grained scoring system has a reported interrater reliability coefficient of .99 and a test–retest reliability coefficient of .95 (Filiatrault-Veilleux et al., 2016b).

#### Receptive Vocabulary

The French version of the Peabody Picture Vocabulary Test–Revised (PPVT-R); the *Échelle de Vocabulaire Image Peabody*, Form A (ÉVIP; Dunn et al., 1993) was used to assess receptive vocabulary. In this test, the child is asked to identify the image named out loud by the experimenter, among a set of four possible images. The test includes 170 items of increasing difficulty. The administration of the test concludes once the child makes six errors within a sequence of eight consecutive items. The raw score is determined by adding up the number of correct responses. This procedure is derived from the original PPVT-R and the terms included in the French version constitute a representative sample of the French language (Pauzé et al., 2004). The reported homogeneity coefficients range from .66 to .88 across different age groups, while the test–retest reliability coefficient is .72 (Dunn et al., 1993).

#### Theory of Mind

An experimental story-based False Belief task was used to assess children's ability to infer behavior on the basis of mental states (Yazdi et al., 2006). The story *Julie et son ballon*, a French translation of a seven-pages long story entitled “Sally,” as described in Yazdi et al. (2006), was read to the children. Participants were presented with one picture shown at a time. The pictures depict Julie playing with a ball, first placing it under her bed and leaving the room. Her father is depicted taking the ball from under her bed and placing it in a toy chest. Four questions are asked to the child and marked as correct or incorrect. After showing the first five images, the child participant is asked two questions regarding the placement of the ball. The questions accompanying the seventh image ask where Julie believes the ball is and where she will look for the ball first. The total score for this task is reported out of four points.

#### Phonological Short-Term Memory

A nonword repetition task (*repetition de logatomes*), derived from the Outil de DÉpistage des DYSlexies (ODEDYS) (Jacquier-Roux et al., 2005), consisting of a list of 20 nonword items, was used as a measure of phonological short-term memory (Coady & Evans, 2008; Talli & Stavrakaki, 2020). The examiner had to say each nonword out loud and the child participants were required to repeat after each item. The nonwords varied in length, from two to five syllables, and were presented in increasing length order. Omissions and substitutions of phonemes were counted as incorrect, whereas distortions did not result in a loss of points as per scoring procedures described in Thordardottir et al. (2011). Total raw score is out of 20 points.

### Data Analysis

Analyses were all performed using SPSS IBM's Statistics 26 for Mac. Standard descriptive statistics (means and standard deviations) were first used to summarize the scores of all four measures for all participants. To address research questions one using group comparisons, analysis of covariance (ANCOVAs) were used to compare the performance of children with DLD to TLD children on measures of inferential comprehension, receptive vocabulary, phonological short-term memory, and ToM, controlling for level of parental education and biological sex. An eta squared value (*η*^2^) was calculated to indicate the effect sizes, with a negligible effect size defined as *η*^2^ < 0.01; a small effect size as 0.01 ≤ *η*^2^ < 0.06; a moderate effect size as 0.06 ≤ *η*^2^ < 0.14; and a large effect size as *η*^2^ ≥ 0.14 (Cohen, 1988). To address research question two, Pearson's r correlations were conducted to examine associations between all variables across the whole sample (DLD and TLD), followed by a hierarchical regression analysis with inferential comprehension performance as the dependent variable, and all other significantly correlated variables as independent concurrent predictors. Cohen's *f*^2^ was used to indicate effect sizes, with *f*^2^ ≥ 0.02 as a small effect size, *f*^2^ ≥ 0.15 as medium effect, and *f*^2^ ≥ 0.35 as large effect sizes (Cohen, 1988; Selya et al., 2012). Both the LPE and diagnosis group variables were organized into dummy variables; with high level of parental education (college or university degree) coded as 1, and low level of parental education (below or high school level) coded as 0. This coding was defined in accordance with low maternal education as an early risk factor for language impairment as described in Stanton-Chapman et al. (2002). For the diagnosis condition, typical language development was coded as 1 and DLD coded as 0. Normality of data was assessed using the Shapiro–Wilk test on all variables. An alpha level of 0.05 was used for all statistical tests.

## Results

### Research Question 1: Group Comparisons (DLD vs. TLD) on Measures of Oral Inferential Comprehension, Receptive Vocabulary, ToM and Phonological Short-Term Memory

**Figure 1.**
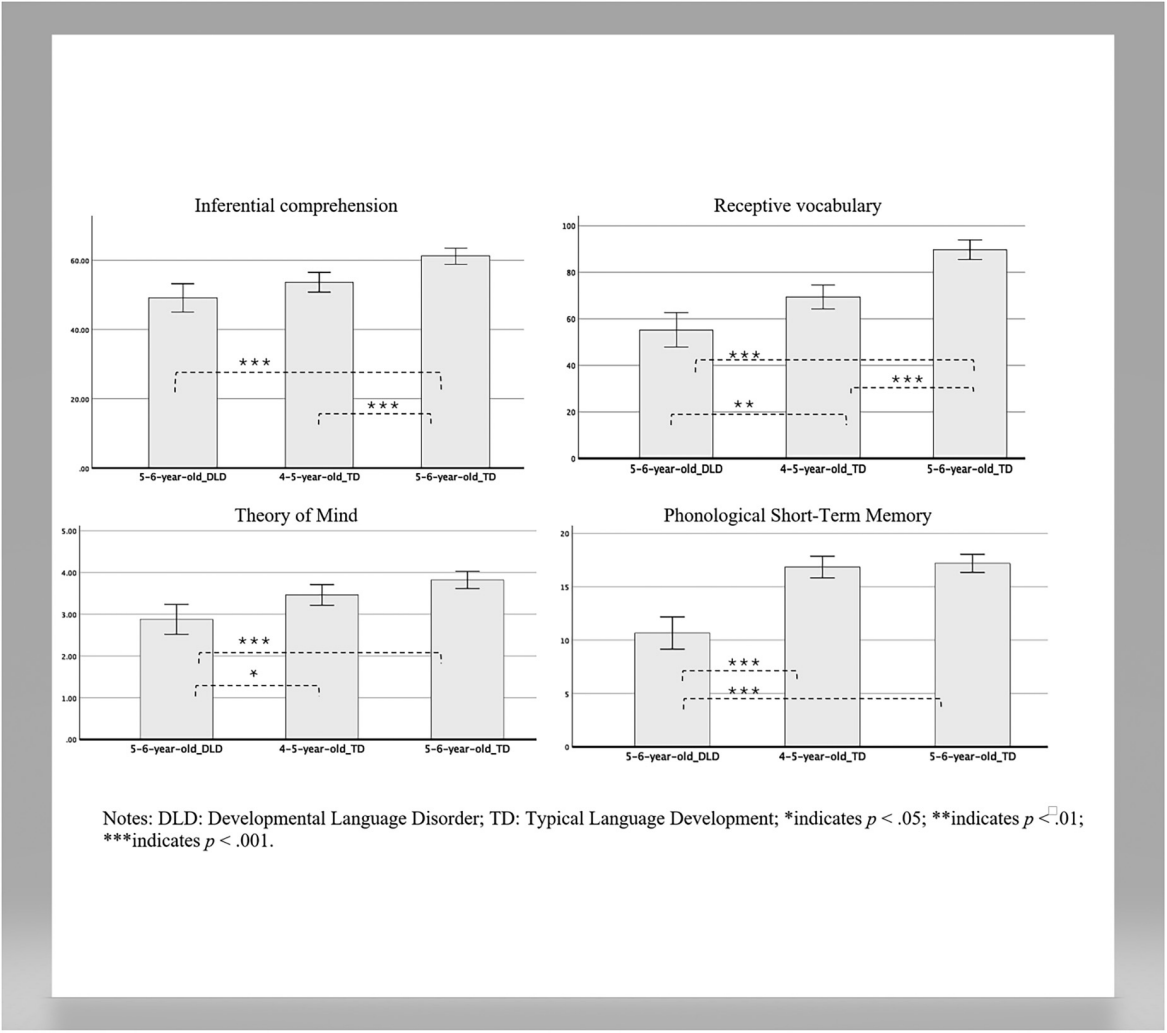
Adjusted means on measures of inferential comprehension (total score), receptive vocabulary (raw score), ToM (total score) and phonological short-term memory (total score), controlled for LPE and biological sex. LPE=Level of Parental Education; ToM= theory of mind.

To answer the first research question, ANCOVAs were performed to compare children with DLD to two groups of TLD children, identifying potential differences between groups on all measures. Table 2 and Figure 1 depict descriptive statistics and performances, including means and standard errors, confidence interval and group differences of preschool children with DLD and both groups of TLD children on measures of inferential comprehension, receptive vocabulary, ToM, and phonological short-term memory in raw scores. ANCOVAs revealed significant group differences on all four dependent variables with large effect sizes, after controlling for LPE and biological sex: inferential comprehension: (*F*(2, 109) = 9.04, *p* < .001, *η*^2^ = .26); receptive vocabulary: (*F*(2, 109) = 20.83, *p* < .001, *η*^2^ = .44); ToM: *(F*(2, 108) = 10.35, *p* < .001, *η*^2^ = .17) and phonological short-term memory: *(F*(2, 108) = 15.64, *p* < .001, *η*^2^ = .38). Post hoc analysis using Bonferroni corrections revealed that children with DLD received statistically significantly lower scores when compared to same-age TLD children (*p* < .001) on all four measures. Moreover, children with DLD obtained significantly lower scores when compared to the younger age group for receptive vocabulary (*p* = .008), ToM (*p* = .028), and phonological short-term memory (*p* < .001), and comparable results for inferential comprehension (*p* = .234). Finally, when looking at both groups of TLD children, younger children performed similarly to children aged 5 to 6 years on measures of ToM (*p* = .092) and phonological short-term memory (*p* = 1.000); and the younger group performed significantly lower on measures of oral inferential comprehension (*p* < .001) and receptive vocabulary (raw scores, *p* < .001) when compared to TLD children aged 5 to 6 years. However, when using standardized scores on the receptive vocabulary task, as expected, both TLD groups’ performances were found to be comparable (*p* = 1.000).

**Table 2.** Descriptive Statistics and Group Differences (ANCOVAs) on All Four Variables, Controlling for LPE and Biological Sex.

Variable	5–6 y.o. DLD *(n* = 21) *M (SE)* *CI*	4–5 y.o. TLD (*n* = 37) *M (SE)* *CI*	5–6 y.o. TLD (*n* = 54) *M (SE)* *CI*	Group comparisons		
				ANCOVA *F*	*η* ^2^_p_	Post hoc comparisons
Inferential comprehension	49.18 (2.06) [45.10, 53.27]	53.69 (1.43) [50.86, 56.51]	61.22 (1.18) [58.88, 63.57]	9.04***	.26	5–6 y.o. DLD = 4–5 y.o.TLD < 5–6 y.o. TLD
Receptive vocabulary	55.20 (3.7) [47.78, 62.62]	69.36 (2.59) [64.23, 74.49]	89.63 (2.15) [85.37, 93.89]	20.83***	.44	5–6 y.o. DLD < 4–5 y.o.TLD < 5–6 y.o. TLD
Theory of mind	2.88 (.18) [2.52, 3.23]	3.46 (.12) [3.22, 3.71]	3.82 (.10) [3.61, 4.03]	10.35***	.17	5–6 y.o. DLD < 4–5 y.o.TLD = 5–6 y.o. TLD
Phonological short-term memory	10.67 (.76) [9.15, 12.18]	16.84 (.51) [15.82, 17.86]	17.19 (.43) [16.34, 18.03]	15.64***	.38	5–6 y.o. DLD < 4–5 y.o.TLD = 5–6 y.o. TLD

*Notes*. Values in brackets indicate the 95% confidence interval (CI). *p < .05, **p < .01, *** *p* < .001. ANCOVA=analysis of covariance; DLD=Developmental Language Disorder; LPE=level of parental education; M=mean; SE=standard error; TLD=Typical Language Development; y.o.=year-old.

### Research Question 2: Contribution of Receptive Vocabulary, ToM, and Phonological Short-Term Memory to Oral Inferential Comprehension Abilities Across the Sample (DLD and TLD)

To answer the second research question, Pearson correlations and hierarchical regression analyses were performed to investigate how ToM, receptive vocabulary, and phonological short-term memory contribute to inferential comprehension across the sample. Table 3 presents the means, standard deviations and bivariate Pearson correlations with confidence intervals for all variables. With specific regards to inferential comprehension, significant positive relationships were found were found with age (in months) (*r* = .38, *p* < .01), LPE (*r* = .26, *p* < .01), diagnosis group (*r* = .36, *p* < .01), receptive vocabulary (*r* = .57,*p* < .01), phonological short-term memory (*r* = .31, *p* < .01), and ToM (*r* = .55, *p* < .01), but not biological sex (*r* = .15, *p* = .15). According to Cohen (1988), effect sizes between inferential comprehension and receptive vocabulary and ToM indicate a large effect, as the other associations indicate medium effect sizes.

**Table 3.** Means, Standard Deviations and Bivariate Pearson Correlations With Confidence Intervals for Preschool Children With and Without DLD.

Variable	*N*	*M*	*SD*	1.	2.	3.	4.	5.	6.	7.	8.
1. Age	112	67.70	9.85	–							
2. Biological sex	112	1.44	.49	−.03 [−.21, .16]	–						
3. LPE	110	.97	.16	−.14 [−.32, .05]	.15 [−.04, .32]	–					
4. Diagnosis group	112	.81	.39	−.29** [−.45, .11]	.24* [.06, .41]	.37** [.19, .52]	–				
5. Receptive vocabulary	112	76.23	21.11	.31** [.13, .47]	.16 [−.03, .33]	.27** [.09, .43]	.52** [.37, .65]	–			
6. Phonological working memory	111	16.04	3.85	−.05 [−.23, .14]	.17 [−.02, .34]	−.02 [−.20, .17]	.54** [.40, .66]	.38** [.21, .53]	–		
7. ToM	111	3.53	.82	.19* [.01, .37]	.11 [−.08, .29]	.04 [−.15, .23]	.36** [.19, .52]	.49** [.33, .62]	.38** [.21, .53]	–	
8. Inferential comprehension	112	56.55	9.79	.38** [.20, .52]	.15 [−.04, .32]	.26** [.07, .43]	.36** [.19, .51]	.57** [.43, .69]	.31** [.13, .47]	.55** [.40, .66]	–

*Notes*: values in brackets indicate the 95% confidence interval for each correlation; *p < .05; **p < .01. According to Cohen (1988), effect size for *r* < .10 indicates a small effect, .11 < *r* > .49 indicates a medium effect, and *r* > .50 indicates a large effect. DLD=Developmental Language Disorder; LPE=level of parental education; ToM= theory of mind.

Then, a hierarchical regression analysis was conducted to predict inferential comprehension from the relative contributions of age, LPE, diagnosis group, receptive vocabulary, phonological working memory, and ToM (Table 4). Age and LPE were entered in step 1 to factor out their effects on inferential comprehension (model 1). This resulted in a significant model, *F*(2, 109) = 17.64, *p* < .001, *R*^2^ = .25, adjusted *R*^2^ = .23, Cohen's *f*^2^ = .33. These factors accounted for 25% of the unique variance, indicating that children's age and children with higher LPE accounted for a significant amount of variability in inferential comprehension. Diagnosis group was entered in step 2 to determine the extent to which the children presenting with DLD predicted inferential comprehension over and above age and LPE (model 2). This resulted in a significant model, *F*(3, 106) = 24.183, *p* < .001, *R*^2^ = .41, adjusted *R*^2^ = .39, Cohen's *f*^2^ = .695. Diagnosis group contributed significantly, explaining an additional 3% of the unique variance in inferential comprehension. Receptive vocabulary, ToM, and phonological short-term memory were entered in step 3 (model 3). This resulted in a significant model, *F*(5, 103) = 23.200, *p* < .001, *R*^2^ = .53, adjusted *R*^2^ = .51, Cohen's *f*^2^ = 1.267, with ToM (*t* = 4.09, *p* < .001) as a significant concurrent predictor, over and above age, LPE and diagnostic group. Receptive vocabulary was close to significance level (*t* = 1.92, *p* = .057) and the contribution of phonological short-term memory was nonsignificant and therefore excluded from the model. Models 2 and 3 indicated a large effect size (Cohen, 1988; Selya et al., 2012). Variance inflation factors (VIF) for all predictors were ranging between 1.23 and 1.99, which suggested no threat of multicollinearity. Overall, the results indicated that when controlling for age, LPE, and diagnosis, children are likely to have better inferential comprehension abilities if they perform well on a measure of ToM.

**Table 4.** Summary of Hierarchical Regression Analysis for Predicting Inferential Comprehension, Using All Potential Variables Across All Participants (*N* = 110–111).

Variable	Model 1					Model 2					Model 3				
	*B*	Std. error	Beta	*t*	*p*	*B*	Std. error	Beta	*t*	*p*	*B*	Std. error	Beta	*t*	*p*
Age	.43	.09	.43	5.01	<.001	.53	.08	.53	6.76	<.001	.33	.09	.33	3.65	<.001
LPE	19.09	5.09	.32	3.75	<.001	10.22	4.84	.17	2.11	.037	9.73	4.50	.16	2.16	.033
Diagnosis group						11.37	2.14	.44	5.32	<.001	5.11	2.53	.19	2.02	.046
Receptive vocabulary											.09	.05	.19	1.92	.057
ToM											3.93	.96	.32	4.09	<.001
*R* ^2^	.234					.406					.530				

*Note*: LPE=Level of Parental Education; ToM= theory of mind.

## Discussion

This study sought to describe oral inferential comprehension abilities, receptive vocabulary, ToM, and phonological short-term memory in 121 young French-speaking preschoolers with and without DLD aged 4 to 6 years. The first research question aimed to answer how young children presenting with DLD aged 5 to 6 years perform when compared to same-age and younger TLD peers on measures of oral inferential comprehension, receptive vocabulary, ToM, and phonological short-term memory. The second question aimed to answer whether receptive vocabulary, ToM, and phonological short-term memory significantly contribute to oral inferential comprehension abilities of young preschool children with and without DLD aged 4 to 6 years of age, after controlling age and LPE.

In response to research question one, after controlling for LPE and biological sex, we found that children with DLD performed significantly below same-age TLD peers on all four measures. Their performance was also found to be comparable to younger children on oral inferential comprehension, and significantly below on receptive vocabulary, phonological short-term memory and ToM. For oral inferential comprehension specifically, these results are aligned with previous research indicating that when answering inferential questions, children with DLD usually obtain comparable results to younger or language-matched children (Dodwell & Bavin, 2008; Dawes et al., 2019; Ford & Milosky, 2008; Filiatrault-Veilleux et al., 2015b) and lower to same-age typically developing peers (e.g., Bishop & Adams, 1992; Botting & Adams, 2005; Norbury & Bishop, 2002). For receptive vocabulary and phonological working memory, our results also align with those of Gray (2006), who observed that 5- to 6-year-olds with DLD performed lower than both age matched typically developing (TD) children and 4- to 5-year-old typically developing children. In a similar vein, it was also reported that children with DLD tend to perform significantly lower on ToM tasks when compared to age-matched children (Andrés-Roqueta et al., 2013; Nilsson & Lopez, 2015; Vissers & Koolen, 2016). Altogether, these results highlight the early difficulties experienced by preschool children with DLD with regard to oral inferential comprehension, receptive vocabulary, phonological short-term memory and ToM and reaffirm the importance of documenting language comprehension difficulties from a young age in this population.

In response to research question two, receptive vocabulary, ToM, and phonological working memory were all found to be significantly correlated with oral inferential comprehension. However, only ToM was found to be a significant concurrent predictor when controlling for age, LPE, and diagnosis condition in a regression analysis. In the final model, age, LPE, diagnosis condition, receptive vocabulary and ToM accounted for 53% of the total variance in inferential comprehension in preschoolers with and without DLD aged 4 to 6 years. Our results also indicate that when controlling for age, LPE and diagnosis condition, children are likely to have better inferential comprehension abilities if they perform well on a measure of ToM. These findings align with Dawes et al. (2018) study, highlighting the importance of ToM skills when answering inferential comprehension questions, which are also in line with previous literature documenting the contribution of false belief understanding to language comprehension abilities of children (e.g., Guajardo & Cartwright, 2016; Desmarais et al., 2016; Dore et al., 2018). Altogether, it underscores the important role of ToM in the development of language abilities, especially oral inferential comprehension.

## Strengths and Limitations

This research sought to provide a better understanding of the language and cognitive skills contributing to oral inferential comprehension in preschool children with and without DLD, notably ToM, receptive vocabulary and phonological short-term memory, while accounting for confounding variables such as LPE, age and biological sex. Such characterization of early listening comprehension profiles of preschoolers with and without DLD can provide crucial information for the design of early targeted interventions for children presenting with language difficulties from a young age. However, some limitations must be acknowledged. First, a higher number of participants in the DLD group would have provided a more robust dataset allowing for further comparisons between and within groups. The relatively small sample size of the DLD group, which constituted a smaller proportion of the total sample, may limit the generalizability of the findings to the broader population of individuals with DLD. Additionally, incorporating a full range of linguistic and cognitive variables as potential contributors to listening comprehension, such as those included in the Direct and Indirect Effects model of Reading (DIER) (Kim, 2020), would have offered a better understanding and a more complete picture of listening comprehension abilities of these children. Similarly, future research should delve deeper in the exploration of social cognition and ToM abilities in children with DLD to better understand the nature of the relationship with listening comprehension. Finally, although significant demographic differences between groups were statistically controlled in the analysis, such as biological sex and LPE, they may still have influenced children's performance and the overall findings of this study.

## Clinical Implications

This study reiterates the early listening comprehension difficulties experienced by preschool children with DLD when compared to TLD children and contributes to a better understanding of early listening comprehension profiles of young children with and without DLD from a young age. Early assessment can enable speech-language pathologists to provide early support for this essential skill, in addition to the language and cognitive skills contributing to oral inferential comprehension difficulties in this population, such as ToM and vocabulary knowledge. Previous studies have illustrated that assessment and intervention at this earlier stage can lead to improvements in inferential comprehension of children, specifically through a scaffolded approach (Dawes et al., 2018; Desmarais et al., 2013; van Kleeck et al., 2006). Moreover, a recent meta-analysis indicates that inferential comprehension instruction is effective for a range of learners, including preschoolers (Rice & Wijekumar, 2024). Due to the overarching effect that inferential comprehension has on a child's educational success (Bishop, 2014; Kendeou et al., 2020; van Kleeck, 2008), having earlier assessment and intervention would help set up children for success in the future. Finally, considering that challenges related to language comprehension are acknowledged to be persistent and less responsive to intervention (Clark et al., 2007; Roberts & Kaiser, 2012), these findings can help inform the development of targeted, evidence-based interventions aiming at supporting language comprehension of young children with DLD.
